# Colonic Pseudo-Obstruction in an Elderly Patient: Resolution Following Correction of Hypokalemia

**DOI:** 10.7759/cureus.96736

**Published:** 2025-11-13

**Authors:** Fahad S Alrashidi, Amer I Albulushi

**Affiliations:** 1 Internal Medicine/Nephrology, Diriyah General Hospital, Riyadh Third Health Cluster, Diriyah, SAU; 2 Internal Medicine, Diriyah General Hospital, Riyadh Third Health Cluster, Diriyah, SAU

**Keywords:** gastro-intestinal symptoms, intestinal pseudo-obstruction, other causes of hypokalemia, potassium disorder, teaching in emergency medicine

## Abstract

Colonic pseudo-obstruction (Ogilvie’s syndrome) is an uncommon but clinically significant cause of large bowel dilatation in the absence of mechanical obstruction. Electrolyte imbalances, particularly hypokalemia, can impair colonic motility and precipitate pseudo-obstruction. We report a case of an elderly male with multiple comorbidities who presented with progressive abdominal distention. Imaging revealed marked colonic dilation without a transition point, while laboratory testing showed severe hypokalemia (2.8 mmol/L). Conservative management, including electrolyte replacement, resulted in marked clinical improvement within 48 hours, following normalization of potassium to 4.0 mmol/L. This case underscores the importance of recognizing and correcting reversible metabolic disturbances before pursuing invasive interventions.

## Introduction

Acute colonic pseudo-obstruction, also known as Ogilvie’s syndrome, is characterized by massive colonic dilatation in the absence of a mechanical cause. It most commonly occurs in elderly or debilitated patients with multiple comorbidities, such as neurological disorders, diabetes mellitus, or cardiovascular disease [1].

The pathophysiology involves autonomic dysregulation leading to impaired colonic motility [2]. Contributing factors include electrolyte abnormalities, infection, trauma, surgery, and certain medications. If not recognized early, the condition can progress to bowel ischemia or perforation [3].

Therefore, identifying reversible precipitating factors, particularly hypokalemia, is crucial for successful conservative management [4].

## Case presentation

An 81-year-old male nursing home resident presented with progressive abdominal distention and mild abdominal discomfort over two days. His baseline functional status included limited mobility and regular bowel movements, as reported by the nursing staff.

His comorbidities included long-standing hypertension, type 2 diabetes mellitus, and a previous ischemic cerebrovascular event with residual mild right-sided weakness. His medications included low-dose aspirin, valsartan, escitalopram, levetiracetam, and atorvastatin, with no recent use of opioids or anticholinergic agents. We considered volume overload, hypoalbuminemia, recent hospitalization, immobility, and electrolyte disturbances as potential contributors to colonic dysmotility; however, apart from hypokalemia and hypomagnesemia, no new or reversible triggers were identified.

On examination, he was afebrile (temperature 36.8°C), with blood pressure 118/64 mmHg, heart rate 78 beats/min, respiratory rate 18 breaths/min, and oxygen saturation 97% on room air. The abdomen was markedly distended but soft and non-tender, with normal bowel sounds and continued passage of stool.

Baseline renal function was preserved (creatinine 95 µmol/L, estimated glomerular filtration rate (eGFR) 38 mL/min/1.73 m²). Serum potassium was severely reduced at 2.8 mmol/L, and magnesium was also low at 0.6 mmol/L, both of which can impair colonic smooth muscle contractility (Table 1).

**Table 1:** Summary of laboratory results with corresponding normal reference ranges. eGFR: Estimated glomerular filtration rate; CKD: Chronic kidney disease.

Test	Result	Normal Range
Potassium	2.8 mmol/L	3.5-5.0 mmol/L
Sodium	139 mmol/L	135-145 mmol/L
Magnesium	0.6 mmol/L	0.7-1.0 mmol/L
Creatinine	95 µmol/L	60-110 µmol/L
Albumin	36 g/L	35-50 g/L
WBC	7.8 × 10⁹/L	4-11 × 10⁹/L
eGFR	38 mL/min/1.73 m²	CKD stage IIIb (30-44.9 mL/min/1.73 m²)

**Figure 1:**
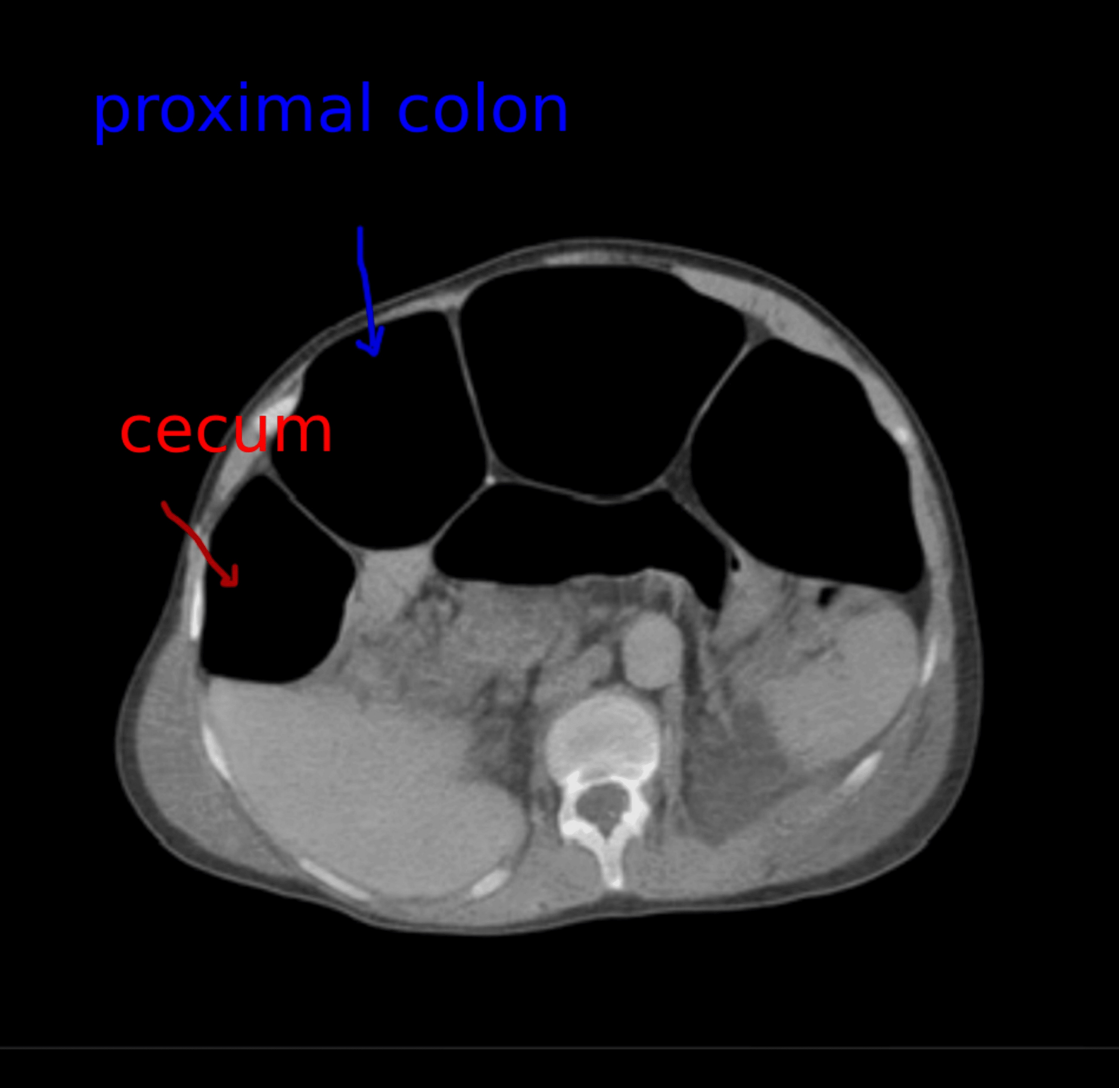
Axial contrast-enhanced CT of the abdomen showing marked gaseous distention of the cecum (red arrow) and proximal colon (blue arrow), measuring approximately 13 cm in diameter, without a transition point, consistent with acute colonic pseudo-obstruction (Ogilvie’s syndrome).

Contrast-enhanced CT scan of the abdomen and pelvis demonstrated marked gaseous distention of the colon, with a maximal diameter of 13 cm at the cecum and proximal transverse colon, with no clear transition point or mechanical obstruction (Figure 1). Mild ascites and small bilateral pleural effusions were also noted. Oral contrast was used, and the radiology report was issued on day 1 of admission.

The patient was managed conservatively with bowel rest (nil per os), nasogastric decompression, and correction of electrolytes. Potassium chloride was administered as 40 mmol oral potassium chloride (KCl) every 6 hours plus 20 mmol intravenous KCl in 0.9% saline over 4 hours, repeated as needed, with continuous cardiac monitoring. Intravenous magnesium sulfate (2 g over 2 hours) was given to correct hypomagnesemia, which can further impair potassium repletion. Over the subsequent 48 hours, serum potassium increased from 2.8 to 4.0 mmol/L, and magnesium normalized to 0.8-0.9 mmol/L.

Over the following 48-72 hours, the patient’s abdominal distention gradually subsided, his discomfort resolved completely, and he resumed regular bowel movements. No endoscopic decompression or neostigmine was required. Repeat cross-sectional imaging was not performed because of the clear clinical improvement and the absence of features suggesting ischemia or perforation.

Clinical improvement in abdominal distention closely coincided with normalization of potassium and magnesium levels, suggesting a strong temporal association between electrolyte correction and resolution of pseudo-obstruction, although causation cannot be definitively established from a single case.

## Discussion

Acute colonic pseudo-obstruction represents a failure of colonic motility due to autonomic dysregulation and/or smooth muscle dysfunction and is an important, albeit uncommon, cause of large-bowel dilatation in hospitalized elderly patients [1,2]. Electrolyte disturbances are well-recognized precipitants, and hypokalemia in particular may be underappreciated [1,2]. Potassium depletion can hyperpolarize smooth muscle cells, decrease excitability, and impair peristaltic activity, especially in the cecum and proximal colon, which are highly sensitive to changes in serum electrolytes [1,2]. Concomitant hypomagnesemia may further impair intestinal smooth-muscle function and hinder potassium repletion [2].

In the present case, severe hypokalemia (2.8 mmol/L) with concurrent hypomagnesemia were the only clearly reversible metabolic abnormalities identified. Following correction of potassium to 4.0 mmol/L and normalization of magnesium over approximately 48 hours, the patient’s abdominal distention and discomfort resolved without pharmacologic or endoscopic decompression. Although other factors such as advanced age, immobility, comorbidities, and chronic medications likely contributed to colonic dysmotility [1,2], the close temporal association between electrolyte normalization and clinical improvement suggests that restoration of potassium and magnesium levels played a major role in the resolution of pseudo-obstruction, rather than a fixed structural or neurological lesion [2].

Typically, neostigmine, an acetylcholinesterase inhibitor that enhances colonic motility, is reserved for patients who do not respond to conservative measures or whose cecal diameter remains markedly dilated (often ≥12 cm) with ongoing symptoms [3-5]. In our patient, careful conservative management with early optimization of potassium and magnesium was sufficient to achieve recovery, obviating the need for neostigmine and thereby avoiding potential adverse effects such as bradycardia and hypotension, which may be particularly hazardous in elderly patients with multiple comorbidities [3-5].

The educational value of this case lies in highlighting that, in selected patients with acute colonic pseudo-obstruction, meticulous evaluation and correction of electrolyte disturbances may avert the need for pharmacologic or invasive interventions [1,2,4,5]. While Ogilvie’s syndrome is well recognized, detailed reports emphasizing resolution associated with electrolyte optimization alone appear relatively infrequent in the literature [2,4,5]. This case therefore reinforces the importance of systematically identifying metabolic and iatrogenic triggers before escalating therapy and of interpreting improvements as a strong temporal association rather than definitive proof of causation [1,2].

Clinicians should remain vigilant for hypokalemia, hypomagnesemia, and other reversible metabolic derangements in elderly or immobilized patients presenting with acute colonic distention [1,2]. Early correction of these abnormalities can facilitate recovery, reduce exposure to higher-risk therapies, and may help prevent progression to ischemia or perforation, while close monitoring ensures timely escalation in those who fail conservative management [3-5].

## Conclusions

This case illustrates that, in selected elderly patients with acute colonic pseudo-obstruction, correction of profound hypokalemia and hypomagnesemia may be sufficient to achieve clinical and radiologic resolution without pharmacologic or endoscopic decompression. Clinicians should actively search for and treat reversible metabolic and iatrogenic contributors while maintaining vigilance for clinical or imaging signs that mandate escalation of care. Larger observational studies are needed to better quantify how often electrolyte correction alone is sufficient and to clarify the relative contribution of hypokalemia versus other risk factors.
